# Systematic review of associations of polychlorinated biphenyl (PCB) exposure with declining semen quality in support of the derivation of reference doses for mixture risk assessments

**DOI:** 10.1186/s12940-022-00904-5

**Published:** 2022-10-11

**Authors:** Sibylle Ermler, Andreas Kortenkamp

**Affiliations:** College of Health, Medicine and Life Sciences, Centre for Pollution Research and Policy, Brunel University London, Kingston Lane, Uxbridge, UB8 3PH UK

**Keywords:** Polychlorinated biphenyl, Semen quality, Reference dose, Mixture Risk Assessment, Male reproduction

## Abstract

**Background:**

Mixture risk assessments require reference doses for common health endpoints of all the chemicals to be considered together. In support of a mixture risk assessment for male reproductive health, we conducted a systematic review of the literature on associations between exposures to Polychlorinated Biphenyls (PCBs) and declines in semen quality. PCBs can act as Aryl-hydrocarbon Receptor (AhR)-agonists and Androgen Receptor (AR)-antagonists, both mechanisms which can affect sperm parameters. PCBs and other AR-antagonists can produce additive combination effects. Based on these observations our objective was to systematically gather data from animal and human studies to derive a reference dose for declines in semen quality for individual PCB.

**Methods:**

We systematically reviewed and evaluated the evidence in human epidemiological and experimental animal studies on associations between PCBs and deteriorations in semen quality. Human data and findings from animal studies with PCB mixtures were considered as supporting evidence. Information for individual congeners from animal studies was required for inclusion in mixture risk assessment. Using a robust confidence rating approach, we identified suitable studies to derive reference doses for individual PCB congeners.

**Results:**

Evaluation of human epidemiological studies revealed several reports of adverse effects on sperm parameters linked to PCB exposures, although some studies reported improved semen quality. Our review of experimental animal studies found that treatments with PCBs affected semen quality, in most cases adversely. We found robust evidence that PCB-118 and -169 were linked to declines in semen quality. Evidence for adverse effects of PCB-126, -132, -149, and -153 was moderate, whereas for PCB-77 it was slight and for PCB-180 indeterminate. Using widely accepted risk assessment procedures, we estimated reference dose values of 0.0029 µg/kg/day for PCB-118 and 0.00533 µg/kg/day for PCB-169. In addition, we derived values for PCB-126: 0.000073 µg/kg/day, PCB-132: 0.0228 µg/kg/day, PCB-149: 0.656 µg/kg/day, and PCB-153: 0.0058 µg/kg/day.

**Conclusions:**

We found robust evidence for links between PCB exposure and deteriorations in semen quality, and derived reference doses for a set of congeners. We intend to use these values in combination with congener-specific exposure data in a mixture risk assessment for declines in semen quality, involving several other antiandrogenic chemicals.

**Supplementary Information:**

The online version contains supplementary material available at 10.1186/s12940-022-00904-5.

## Introduction

Polychlorinated biphenyls (PCBs) are a group of organic chlorine compounds which were widely used as technical mixtures in building materials and electrical equipment. The group consists of 209 congeners exhibiting a variety of toxic effects, depending on their structure. PCBs are classified as persistent organic pollutants (POPs) and due to their toxicity they have been banned under the Stockholm Convention on Persistent Organic Pollutants in 2001 [1]. However, owing to their persistence and wide distribution, they are still present in the environment and human tissues.

Humans are exposed to PCBs mainly via the diet, and to a much lesser extent via inhalation or dermal contact. The European Food Safety Authority (EFSA) found the main route of exposure to be food of animal origin with a high fat content such as meat, dairy products and fatty fish [2, 3].

Individual PCB congeners and technical mixtures can act as endocrine disrupting chemicals (EDCs). They are able to interact with several nuclear receptors, including the Aryl hydrocarbon Receptor (AhR), the Androgen Receptor (AR), Constitutive Androstane Receptor (CAR), Pregnane Xenobiotic Receptor (PXR) complex and several others [2–4]. Both dioxin-like (dl) and non-dioxin-like (ndl) PCBs can activate the AhR in vitro [5], while AR antagonism is mainly exhibited by ndl-PCB congeners [6, 7]. Both AhR agonism and AR antagonism can affect male reproductive development in vivo, with effects on sperm quality, regulation of sex hormones and development of reproductive organs [2, 3, 8]. There is epidemiological evidence that exposure to several PCB congeners is associated with adverse male reproductive health outcomes, including cryptorchidism, late pubertal onset and deteriorations of semen quality [2].

Due to their ubiquitous distribution in the environment and human tissues, exposure is not to any single congener, or even PCBs alone. Instead, we are exposed to a range of chemicals which can interfere with male reproductive development. Experimental studies have demonstrated that antiandrogenic PCB congeners can produce additive effects in combination with other AR antagonists in vitro [9]. Numerous other chemicals are known to affect normal male reproductive development via multiple pathways, initiated by AR antagonism or AhR agonism [8]. These include bisphenol A (BPA), phthalates, parabens, dioxins, polybrominated diphenyl ethers (PBDEs), some azole pesticides and analgesics [8]. Some of these EDCs have been demonstrated to produce combination effects interfering with male reproductive development in vivo, with observed effects comprising retained nipples in male offspring [10] as well as deteriorations in semen quality [11]. In addition to their ability to produce mixture effects, exposure to these chemicals is also widespread [2, 12–16] and we know that co-exposures to some or all of these chemicals occur [17]. It is plausible that PCB exposures can contribute to such mixture effects. Therefore, mixture effects of chemicals impacting male reproductive health and the accompanying risks call for a systematic investigation, including the contribution of PCB congeners.

Assessment of the combined risk from exposures to several chemicals can be conducted using the Hazard Index (HI) approach [18]. The HI is the sum of Risk Quotients, i.e. the ratio of exposure and a reference dose or health-based guidance value (HBGV) for specific toxicities of individual chemicals included in the assessment. The HI is assessed against a reference value of 1 and values above 1 indicate the fold-exceedance of “acceptable” combined exposures. It is important to select reference doses for similar, or even identical toxicity endpoints to reduce uncertainty and achieve higher consistency in the assessment. Alternatively, it is also possible to evaluate mixture risks by employing relative potency factors (RPF) to express exposures to relevant chemicals in terms of equi-effective fractions of exposures to a reference chemical. This approach is familiar from evaluations of dioxin toxicities in terms of 2,3,7,8-TCDD equivalents. However, both the HI method and the derivation of RPF require the estimation of reference doses for specific toxicities. With the HI, these reference doses are used to build Risk Quotients, and with RPF, they are employed to derive the RPF.

PCBs have been evaluated by EFSA as part of separate assessments for dl-PCBs [2] and ndl-PCBs [3]. The dl-PCBs were assessed together with polychlorinated dibenzodioxins (PCDDs) and polychlorinated dibenzofurans (PCDFs), and a tolerable weekly intake (TWI) of 2 pg WHO_2005_-TEQ/kg was derived for the group of compounds [2]. Whilst the critical toxicity for the TWI was a decline in semen quality, this was based on the epidemiological evidence for dioxins and there were considerable uncertainties regarding the values for the dl-PCBs. For ndl-PCBs, critical toxicities comprise a variety of health endpoints and no health-based guidance values have been established [3]. Overall, references doses for PCBs are either not suitable or not available for inclusion in a mixture risk assessment of declining male reproductive health. To derive reference doses for individual PCB congeners, there is a need to search for suitable studies examining the link between PCB exposure and declines in semen quality.

In this systematic review, we searched the literature for studies investigating PCBs and male reproductive toxicity. We concentrated on declines in semen quality to align our systematic review with current trends observed in Western countries [19]. Semen quality is closely linked to male fecundity [19, 20] and frequently assessed in human and animal studies. Therefore, we chose adverse effects on semen quality as outcome. As our focus was on endocrine mechanisms, we defined declines in semen quality in terms of changes in sperm parameters such as count, concentration, motility, morphology or vitality, the basic semen examination parameters based on WHO guidance and on OECD test guidelines [21, 22]. Sperm DNA damage or aneuploidy were not considered as these are indicative of other mechanisms such as oxidative stress, chromatin packaging abnormalities, and apoptosis [23]. To derive references doses, i.e. exposures no longer associated with declines in semen quality, we were particularly interested in toxicity data for individual PCB congeners as this is required to calculate Risk Quotients in combination with exposure data for individual congeners.

The overall objective of this systematic review was to gather data from animal studies and human epidemiological studies to address the following separate but related questions: what is the strength of evidence of associations between exposure to specific PCB congeners and declines in semen quality? What are the reference doses for specific PCB congeners for semen quality deterioration that can be used in a mixture risk assessment of male reproductive health, with a specific focus on semen quality?

## Materials and methods

### Systematic review

#### Literature search and screening

The methods for the literature search and screening, the study evaluation, data extraction and evidence synthesis are described in detail in the systematic review protocol [24] developed following the COSTER recommendations [25]. In brief, experimental and epidemiological studies examining PCB exposures and declines in semen quality were identified by conducting literature searches in PubMed, Web of Science and Scopus until November 2020. Citation searches of key papers were also conducted. We used the PECO principle for inclusion of animal studies (Populations: laboratory mammalian species; Exposures: PCBs by oral gavage, drinking water or diet; Comparators: animals not exposed to PCBs; Outcomes: semen quality parameters, supplementary table 1) and human studies (Populations: men of reproductive age; Exposures: PCBs, measured as blood, serum or plasma levels; Comparators: men not exposed to PCBs or with PCB levels in lower quartiles; Outcomes: semen quality parameters, supplementary table 2).

The literature review process was coordinated and managed using the freely available online tool CADIMA (https://www.cadima.info/index.php/area/evidenceSynthesisDatabase).

Briefly, and as detailed by Ermler and Kortenkamp, we included experimental studies with laboratory animals that analysed sperm parameters such as total sperm count, concentration, motility, morphology or vitality as outcome measures, which were considered indicative of semen quality [24]. These parameters were selected as they are the basic semen examination parameters according to the standard WHO laboratory manual for the examination of human semen [21]; and are also listed as parameters to be assessed in OECD TG 443 (Extended one-generation reproductive toxicity study for test in of chemicals, [22]). Sperm DNA damage or aneuploidy as well as fertility outcomes were not considered. We excluded studies with non-mammalian species. Data from studies where PCBs were administered during the sensitive window of exposure for male reproductive toxicity (gestational day (GD) 7 to postnatal day (PND) 10) was preferably used, but in the absence of gestational exposure studies, data from postnatal, juvenile, or adult animals were also considered. We included studies that delivered PCB congeners to experimental animals by the intraperitoneal (i.p.) route, as the pharmacokinetics of compounds administered by this route are similar to oral administration, in terms of absorption, metabolism and distribution [26]. In addition, we considered subcutaneous (s.c.) administration to support the evidence for associations between semen quality and PCB exposures but excluded s.c. delivery from derivation of a reference dose due to differently affected toxicokinetics by this route. The full eligibility criteria for animal studies are listed in supplementary table 3.

We incorporated epidemiological studies among adult men (between 18 and 40 years of age) that reported semen quality parameters (total sperm count, sperm concentration, motility, morphology or vitality). Studies on DNA damage or aneuploidy in sperm were excluded as these are not related to reproductive toxicity via endocrine factors. Case–control studies, cohort studies and cross-sectional studies were considered, but we excluded case reports and reviews. Only studies that measured PCB concentration in blood, serum or plasma were included. Measurements in other matrices such as seminal plasma or adipose tissue were not considered. The full eligibility criteria for animal studies are listed in supplementary table 4. The key data extraction elements to summarise study design, experimental model, methodology and results for human and animal studies are provided in supplementary table 5.

#### Study evaluation

Briefly, and as detailed by Ermler and Kortenkamp, we assessed the internal validity of the studies using separate criteria for animal studies and human epidemiological studies [24]. The main concerns were the risk of bias (RoB, i.e. factors affecting magnitude or direction of an effect) and insensitivity (i.e. factors the limit the ability to detect an effect which is actually present).

We appraised the internal validity of animal studies using a risk of bias (RoB) assessment based on a protocol defined for BPA studies by EFSA [27, 28] and further developed in a protocol to appraise animal studies on declining semen quality associated with exposure to BPA [29] or PBDEs [30]. We utilised the NTP OHAT RoB Tool [31], which we adapted further to evaluate the studies we identified for PCBs and semen quality. The key elements of assessment included exposure characterisation (including purity and stability of test compounds, and absence of contaminations), outcome assessment (blinding of the outcome assessors) and power of detecting effects (sufficient number of animals per dose group). Due to the nature of the effects we additionally included a key element for laboratory proficiency (use of a reliable and sensitive animal model and inclusion of a positive control). The use of phytoestrogen-free chow (i.e. soy-free feed) was also considered to be relevant for examinations of semen quality. Accordingly, we included this aspect in the RoB assessment in the additional assessment elements. A detailed list of all the elements of the RoB assessment can be found in the systematic review protocol [24].

Each RoB element was evaluated using the NTP OHAT scores: +  + *definitely low risk of bias*; + *probably low risk of bias*; ~ *probably high risk of bias*; ~  ~ *definitely high risk of bias*. We used a tiered system to rate the studies, adopted from the system described by EFSA [28]. This comprises three tiers, and each study was allocated to one tier as follows: *TIER 1 – high confidence,* where all key elements were scored + or +  + AND no more than one additional question was scored ~ or ~  ~ ; *TIER 2 – medium confidence* was assigned to all combinations not covered by *TIER 1* or *3*; the lowest tier, *TIER 3 – low confidence* was used when any one of the key elements was scored ~ or ~  ~ OR more than 50% of the additional questions were scored ~ or ~  ~ . The RoB assessment protocol is shown in the published protocol, together with instructions how to rate each element of the protocol in terms of the risk categories [24].

We assessed the epidemiological studies of associations between PCB and semen quality using the procedures detailed by Radke et al., with evaluations of exposure measurement, outcome measurement, participant selection, confounding and analysis [32]. The criteria detailed in Radke et al. and listed in the published protocol [24] were applied to judge the quality of each study with respect to its suitability for hazard identification by reaching a consensus in each evaluation domain with the categories *Good*, *Adequate*, *Poor*, or *Critically Deficient*. We then combined the ratings for each evaluation domain to determine an overall study confidence rating of *High*, *Medium*, *Low*, or *Uninformative*.

#### Data synthesis

We summarised the findings and characteristics of the eligible studies in a narrative synthesis. The data synthesis included summaries of PCB exposure ranges not associated with declines in semen quality in animal studies as concluded from the published derived no observed adverse effect levels (NOAELs) or lowest observed adverse effect levels (LOAELs). Only studies we rated as high or medium confidence (*TIER 1* and *TIER 2*) were included in the summary. Studies that were assigned to *TIER 3* were not further analysed in detail. Human studies were qualitatively assessed to compare findings from animal studies with epidemiological evidence.

#### Evidence synthesis

We synthesised the evidence from animal and human studies, using frameworks previously devised for BPA and phthalates and adapted for PCBs [28, 32]. We performed the evidence synthesis for animal and human studies separately.

The evidence from animal studies was categorised as *Robust* if multiple studies with a *TIER 1* confidence rating showed similar adverse effects. Any evidence that cannot be explained by study design or difference in animal model is from studies of lower confidence, *TIER 2* or *TIER 3*. The evidence was rated as *Moderate* when it was insufficiently strong for *Robust*, but contained at least one *TIER 1* study and additional information supporting the findings. The rating of *Slight* was used in circumstances where studies suggested a possible decline in semen quality, but with weak or conflicting findings. *Indeterminate* was given for inconsistent, weak or conflicting findings. We assigned *Compelling evidence of no effect* when studies with high confidence ratings consistently demonstrated a lack of biological effects across species, sexes and exposure levels.

Evidence synthesis for human studies was carried out using the framework established by Radke et al. [32] and adapted for PCBs. The framework assigns the conclusions from the strength of evidence assessment to *Robust, Moderate, Slight, Indeterminate* and *Compelling evidence of no effect*. *Robust* is assigned for evidence from high or medium confidence independent studies that report an association between PCB exposure and declines in semen quality, with reasonable confidence that alternative explanations, including chance, bias, and confounding, can be ruled out across studies. *Moderate* describes a situation with a smaller number of studies (but at least one high or medium confidence study with supporting evidence), with some heterogeneous results, that do not reach the degree of confidence required for robust. *Slight* is used when there are one or more studies reporting an association between PCB exposure and declining semen quality, but considerable uncertainty exists (the evidence is limited to consistent low confidence studies, or higher confidence studies with unexplained heterogeneity). *Indeterminate* describes the situation when either no studies are available in humans or when the evidence is highly inconsistent and primarily of low confidence. *Compelling evidence of no effect* requires several high confidence epidemiological studies reporting null results.

The overall weight of evidence from human and experimental studies was assessed by comparing the findings of the separate evidence synthesis of animal and human data. This was ideally achieved on an individual PCB-congener basis, but where this was not possible, the overall support of animal data by human evidence was considered.

### Derivation of a reference dose for individual PCB congeners for declines in semen quality

We derived a reference dose for individual PCB congeners following the procedure used by EFSA for other toxicity endpoints [2] and previously applied to derive reference doses for PBDEs associated with declines in semen quality [30]. Eligible studies that allowed estimation of a Point of Departure (PoD) were considered for the derivation of a reference dose. The PoDs under consideration were NOAELs or benchmark dose levels (BMDLs). In cases where available data only allowed the estimation of a LOAEL, the NOAEL was extrapolated using a standard assessment factor (AF = 3).

To extrapolate values from rodent studies to humans, we had to consider that PCBs are persistent compounds which bioaccumulate in tissues and can exhibit different kinetic properties in different species. We scaled the doses across different species using the body burden approach as previously described to derive HBGVs for dioxins and dl-PCBs [2, 33]. We employed this approach to estimate rodent body burdens of PCB congeners associated with PoDs for semen quality (“critical” body burden), which were used to derive human intake estimates which would lead to a human body burden equivalent to the critical body burden in rodents.

First, we estimated the body burden at the experimental PoD in the animal study. For studies which used a single oral PCB dose, the body burden was derived by multiplying the PoD with the fraction of the compound absorbed into the animal body (Eq. 1). The absorbed fraction was derived from the oral absorption of the compound. For repeat administration studies, the body burden at the end of treatment was estimated by taking account of the absorption as well as the half-life of the chemical in the animal body. All kinetic parameters were collected from EFSA [2, 33] or published literature [34–37].

1 $${BB}_a=F_{abs,a}\cdot {PoD}$$

with BB_a_ = body burden in the animal (amount/kg bw); F_abs,a_ = fraction of chemical which is absorbed into the animal body; and PoD = point of departure, such as BMDL or NOAEL.

In a second step, we estimated the equivalent human daily intake (EHDI) by using the assumptions outlined in the EFSA opinions on dioxins and dl-PCBs [2, 33] as well as ndl-PCBs [3]. Accordingly, we used a one compartment model to calculate the EHDI by multiplying the animal body burden derived in step one (Eq. 1) with the rate constant for the elimination from humans, divided by the fraction of compound absorbed into the human body (Eq. 2).

2 $$EHDI=\frac{{BB}_a\cdot k_{el,h}}{F_{abs,h}}$$

with k_el,h_ = rate constant for removal from human body (1/day) and F_abs, h_ = Fraction of chemical absorbed into the human body. In the one compartment model k_el,h_ can be calculated according to Eq. 3.

3 $${k}_{el,h}=\frac{ln2}{{t}_{{}^{1}\!\left/ \!{}_{2}\right. , h}}$$

with t_1/2,h_ = halflife of excretion in humans. After substituting k_el,h_ in Eq. 2 with Eq. 3 the EHDI was calculated according to Eq. 4.

4 $$EHDI=\frac{{BB}_a\cdot \mathrm{ln}2}{{t}_{{}^{1}\!\left/ \!{}_{2}\right. , h}\cdot {F}_{abs, h}}$$

An additional AF factor to account for inter-species differences was then applied by dividing the EHDI with 2.5 to derive the reference dose for the individual PCB congener [38]. The toxicokinetic parameters for the PCB congeners for which a reference dose was derived are provided in supplementary table 6.

### Calculation of risk quotients and the HI for selected PCB congeners

We calculated the Risk Quotients for PCB congeners for which we derived a reference dose and where data for exposure via food from the European Union were available. To reflect average and high exposure scenarios, we extracted mean and 95^th^ percentile LB intakes for European adults for PCBs-118, -126 and -169 [2]. For PCB-153 we used the fact that PCB levels in food are highly correlated and thus assumed three times the value for PCB-118 as a worst-case estimate [3]. No exposure data for PCB-132 and -149 were available. We calculated the Risk Quotients for PCBs-118, -126, -153 and -169 by dividing the food intake levels by the derived reference doses. Exposure data for average and high exposure levels are provided in Table 5. The Risk Quotients were then summed up to calculate the HI. Risk Quotients and HI were estimated for both, an average and high exposure scenario.

## Results

**Fig. 1:**
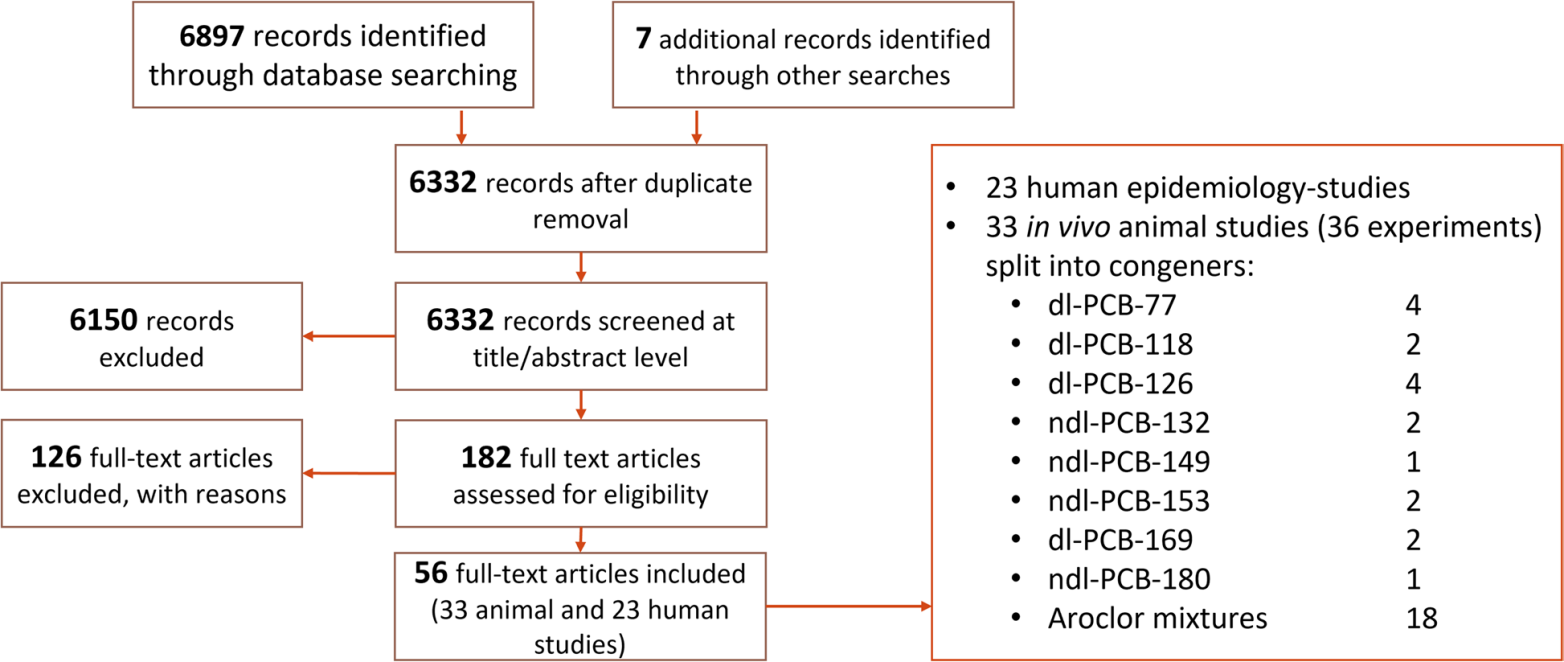
Literature search flow diagram for experimental animal studies and human epidemiological studies of PCB exposures and semen quality

The literature selection process for animal and human epidemiological studies for this systematic review is shown in Fig. 1. Following selection, evaluation and RoB analysis of animal (Tables 1 and 2) and human studies (Table 3), we assessed the strength of evidence for an association between declines in semen quality and experimental exposure to individual PCB congeners in animal studies (Table 2) and population exposure in human epidemiological studies (Table 3). Next, we used data from eligible studies to derive a reference dose for declines in semen quality (Table 4).

**Table 1:**
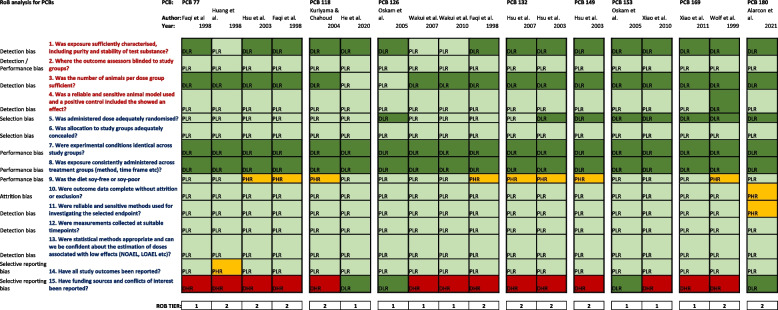
Outcome of Risk of Bias (RoB) analysis for PCBs 77, 118, 126, 132, 149, 153, 169 and 180

Shown is the scoring for each Risk of Bias (RoB) element for the selected animal studies. Questions in red represent key element, questions in dark blue are the remaining elements. The studies were rated as follows: definitely low risk of bias, DLR, in dark green; probably low risk of bias, PLR, in light green; probably high risk of bias, PHR, in yellow; definitely high risk of bias, DHR, in red. The RoB Tier assigned to each study is shown at the bottom. More information on the elements of the RoB is provided in the systematic review protocol [24].

**Table 2:**
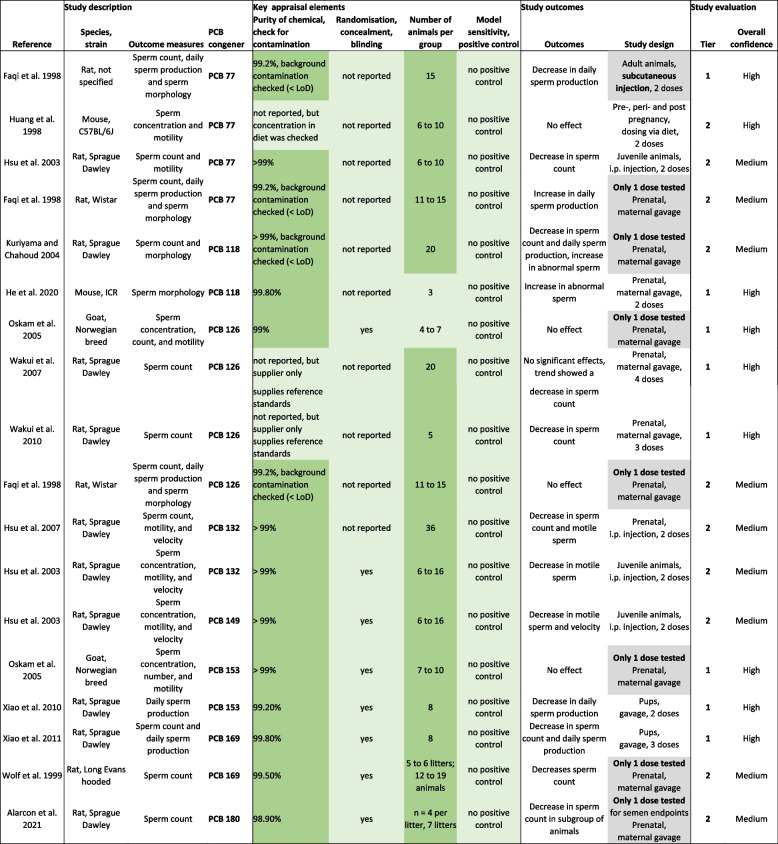
Evaluation of experimental animal studies and semen quality and additional male reproductive endpoints after treatment with PCBs

Colours: Key appraisal elements – Dark green: definitely low risk; light green: probably low risk; light red: probably high; dark red: definitely low risk (note that all elements were definitely or probably low risk). Study outcomes – Grey: admitted as evidence, but not considered for derivation of a reference dose

**Table 3:**
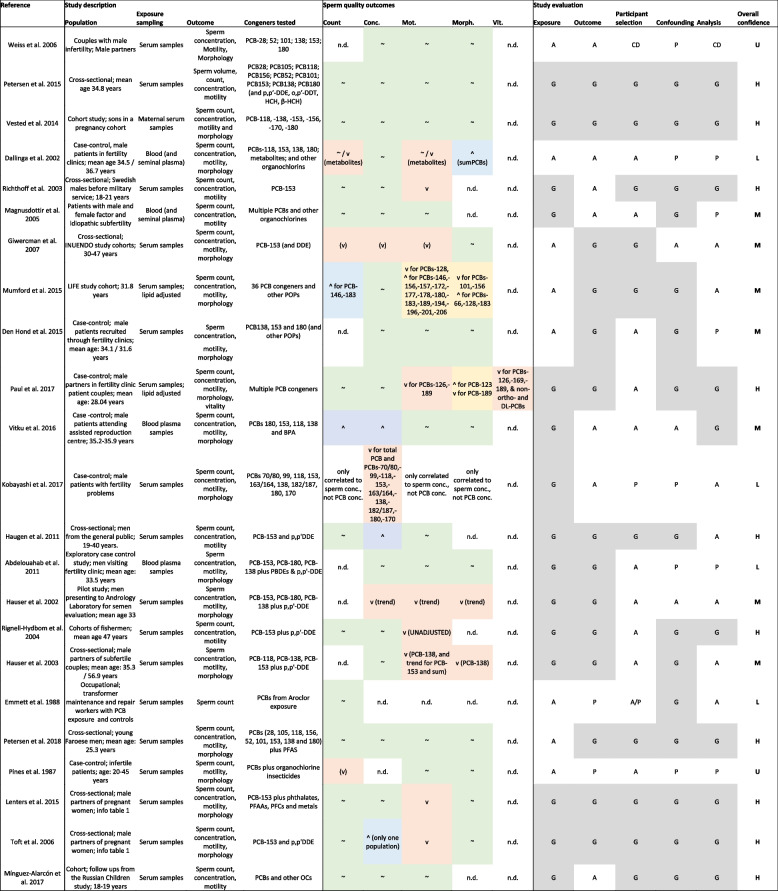
Study evaluation and overall confidence rating of human epidemiological studies of associations of exposures to PCBs with semen quality

*Abbreviations*: Semen quality outcomes – *Conc* Concentration, *Mot* Motility, *Morph* Morphology, *Vit* vitality, v (red shading): decline, ~ (green shading): no association, ^ (blue shading): improvement, v^ (yellow shading): direction of response dependent on congener, *n.d.* Not determined. Study evaluation – *CD* Critically deficient, *P* Poor, *A* Adequate, *G* Good (grey shading). Overall confidence – *U* Uninformative, *L* Low, *M* Medium, *H* High

**Table 4:** Reference doses derived from rodent studies that full-filled all inclusion criteria and passed RoB assessment using the body burden approach

Congener /Study	Tier	Species	LOAEL (µg/kg/d)	NOAEL (µg/kg/d)	BB at NOAEL (µg/kg/d)	EHDI (µg/kg/d)	RfD (µg/kg/d)
PCB-118 He et al. 2020	1	Mouse	20 ^a)^	*6.67* ^a)^	35.5	0.00725	**0.0029**
PCB-126 Wakui et al. 2010	1	Rat	0.25 ^a)^	0.025 ^a)^	0.154	0.00018	**0.000073**
PCB-132 Hsu et al. 2007	2	Rat	1000 ^b)^	*333.33 *^b)^	300	0.0570	**0.0228**
PCB-132 Hsu et al. 2003	2	Rat	9600 ^b)^	*3200* ^b)^	2880	0.547	0.219
PCB-149 Hsu et al. 2003	2	Rat	96,000 ^b)^	9600 ^b)^	8640	1.641	**0.656**
PCB-153 Xiao et al. 2010	1	Rat	2500 ^a)^	25 ^a)^	111	0.0147	**0.00586**
PCB-169 Xiao et al. 2011	1	Rat	25 ^a)^	*8.33 *^a)^	51.2	0.0133	**0.00533**

The reference doses chosen for mixture risk assessment are shown in bold

The NOAEL values shown in italics are extrapolations from studies where only a LOAEL, but no NOAEL was observed. A NOAEL was extrapolated by dividing the LOAEL by a factor of 3

*LOAEL* Lowest observed adverse effect level, *NOAEL* No observed adverse effect level, *BB* Critical body burden, *EHDI* Estimated human daily intake associated with rodent BB at NOAEL, *RfD* Reference dose derived by dividing the EHDI by 2.5

^a)^Repeat administration, BB estimated taking absorption and excretion into account

^b)^Single administration

### Strength of evidence: experimental studies in laboratory animals

#### Study selection and evaluation

Overall, we identified 33 publications that assessed links between semen quality in vivo and exposure to PCBs (Fig. 1). Of these, 15 publications reported on declines in semen quality in vivo upon treatment with individual PCB congeners. Because some studies examined two PCB congeners, we extracted data for a total of 18 separate experimental observations for individual congeners (Table 2). The studies were conducted in rats, mice or goats. We identified four studies examining the effects of PCB-77 [39–42], two studies on those of PCB-118 [43, 44], four studies which looked at PCB-126 [39, 45–47], two studies for PCB-132 [48, 49], one study for PCB-149 [48], two studies reporting on PCB-153 [45, 50], two on PCB-169 [51, 52] and one report on PCB-180 [53]. All these studies were selected for the data extraction process.

A detailed summary of all risk of bias assessments and confidence ratings of these studies is shown in Table 1 and the study evaluations for all individual PCB congeners are summarised in Table 2.

An additional 18 studies which described the effects of PCB mixtures, commercial (Aroclor 1242, 1254, and 1260) or other PCB mixtures were identified. We did not fully evaluate the 18 studies which tested the effects of PCB mixtures (Aroclor 1242, 1254, and 1260 or 1:1 PCB-101/-118) because they were unsuitable for derivation of congener-specific reference doses. However, we summarise their findings in support of the overall evidence. Two studies in mice tested 1:1 mixtures of PCB-101 and -118 and both found decreases in sperm viability [54, 55]. Three studies reported increases in daily sperm production upon treatment with Aroclor mixtures, two of those tested Aroclor 1242 [56, 57] and one Aroclor 1242 and 1254. Two studies of Aroclor 1254 in rats observed no effects on the examined semen parameters [58, 59]. The remaining 11 studies all observed adverse effects upon treatment with Aroclor. Only one tested Aroclor 1260 in rats, and reported decreases in sperm count, motility and daily sperm production [60]. All others tested Aroclor 1254 and adverse effects on various sperm parameters, including number, concentration, motility and morphology were reported [60–65]. Furthermore, Aroclor 1254 was used to induce declines in semen quality to test beneficial co-exposures in five studies [66–70].

We evaluated the internal validity of the 18 experimental observations from the 15 studies which investigated individual congeners by carrying out a risk of bias analysis. All the studies met the key appraisal elements with a rating of “probably low” or “definitely low risk” (Table 1). None of the studies were disqualified due to failure of other elements. The only element which received rankings of “definitely high risk” was inadequate reporting on funding sources and conflicts of interest (14 studies). “Probably high risk” was assigned to the 8 studies that used soy containing diets, and due to a lack of information on attrition and detection in one study (Table 1).

#### Congener-specific studies

##### ***PCB-77***

Of the four studies examining PCB-77, three were conducted in rats [39, 40, 42] and one in mice [41]. All four studies were rated as “probably low” or “definitely low risk” in all key elements, and one had only one “definitely high risk” for another element and was assigned to *TIER 1*, or *High* confidence [40]. The other three had “probably high” or “definitely high risk” ratings in two of the remaining elements and thus were assigned to an overall *Medium* confidence (*TIER 2*) [39, 41, 42]. One rat study which found an increase in daily sperm production upon treatment with PCB-77 only tested one PCB dose (0.1 mg/kg/d at GD15 via maternal gavage) and was therefore excluded from consideration as a basis for deriving a reference dose [39]. The other rat study from this group established a decrease in daily sperm production with a LOAEL of 18 mg/kg/d [40]. However, this study used s.c. injection of PCB-77 and was therefore only included as evidence for a link between PCB-77 and reduced semen quality but not considered for derivation of a reference dose. The only mouse study did not report on the purity of the compound, but was still considered *TIER 2* because PCB-77 was analytically confirmed in the treatments [41]. This study found no effect on semen quality. Finally, Hsu et al. reported a decline in semen quality upon i.p. injection of PCB-77 (NOAEL = 2 mg/kg/d) and was considered for derivation of a reference dose [42].

##### ***PCB-118***

Of the two studies of PCB-118, one was conducted in the rat [43], and the other in mice [44]. We rated the key elements of the study by Kuriyama and Chahood as “probably low risk” or “definitely low risk”, but failed some of the additional elements and therefore assigned an overall *Medium* confidence (*TIER 2*) [43]. The mouse study was “definitely low” or “probably low risk” in all elements and was therefore considered *TIER 1* or of *High* confidence [44]. Both studies reported a decline in semen quality. However, the rat study only tested one dose of PCB-118 (0.375 mg/kg/d) [43] and was thus not taken forward for reference dose derivation. The mouse study which reported a LOAEL of 0.02 mg/kg/d [44] was used to derive a reference dose for PCB-118.

##### ***PCB-126***

There were four studies of PCB-126, of which one was conducted in goats [45] and the other three in rats [39, 46, 47]. The goat study and two of the rat studies were rated at an overall confidence level of “*High*” (*TIER 1*) due to all elements being evaluated as “definitely low” or “probably low risk” [45–47]. The third rat study was evaluated as “definitely low” or “probably low risk” in the key elements but had some other elements rated lower and was thus assigned to *TIER 2*, *Medium* confidence [39]. The goat study [45] and one rat study [39] both reported no effect of PCB-126 on semen quality, however, both studies also only tested one dose and would not have qualified for derivation of a reference dose. Of the other two studies one did not show significant effects, but a trend towards declining semen quality [46]. These trends were confirmed in a later study by the same group after including higher doses, and we used their NOAEL of 2.50E-05 mg/kg/d to derive a reference dose [47].

##### ***PCB-132***

Both studies we identified for PCB-132 were conducted in rats [48, 49] and were evaluated as “definitely low” or “probably low risk” in the key elements but had some other elements rated lower and were therefore considered to be of *Medium* confidence (*TIER 2*). Both studies used i.p. injection of PCB-132 and reported declines in semen quality. One study was conducted in juvenile rats and determined a LOAEL of 9.6 mg/kg/d [48] whereas the second studied prenatal exposure to PCB-132 (LOAEL = 1 mg/kg/d) [49]. Both studies were considered for derivation of a reference dose.

##### ***PCB-149***

The only available study on PCB-149 was evaluated as “definitely low” or “probably low risk” in the key elements but had other elements rated lower and was assigned to *TIER 2* (*Medium* confidence) [48]. This study was conducted in juvenile rats, used i.p. injection of PCB-149 and estimated a NOAEL of 9.6 mg/kg/d which was used to derive a reference dose.

##### ***PCB-153***

Of the two studies reporting on PCB-153, one was conducted in goats [45] and the other in rats [50]. The goat study was evaluated as “definitely low” or “probably low risk” in all elements and rated at an overall confidence level of “*High*” (*TIER 1*) [45]. The rat study was also assigned to *High* confidence (*TIER 1*) as only one additional element was rated lower [50]. The goat study [45] did not find any effects of PCB-153 on semen quality and it also tested only one dose and would not have qualified for derivation of a reference dose. The rat study was conducted in pups and reported a NOAEL of 0.025 mg/kg/d [50] which was used to derive a reference dose.

##### ***PCB-169***

The two studies that examined associations between declines in semen quality and PCB-169 exposure were conducted in rats [51, 52]. Both were rated as “definitely low” or “probably low risk” in the key elements. One had only one additional element rated at definitely high risk and was therefore of overall *High* confidence (*TIER 1*) [51]. The second study was rated lower at two other elements and was thus assigned to *TIER 2* (*Medium* confidence) [52]. This study examined prenatal exposure to PCB-169 exposure and found declines in semen counts [52]. However, it tested only one dose (1.8 mg/kg/d) and was thus not used to derive a reference value. The second rat study used neonatal exposures and reported declines of semen quality with a LOAEL of 0.025 mg/kg/d [51] which was used to derive a reference value.

##### ***PCB-180***

PCB-180 was orally administered to rats during gestation [53]. We assessed this study as “definitely low” or “probably low risk” in the key elements, but lower in other elements and thus assigned to *Medium* confidence (*TIER 2*). However, the focus of the study was on other endpoints and declines in sperm counts were only observed in three out of seven animals, and only in those with damage to the seminiferous tubule sperm counts. Furthermore, sperm counts were only assessed at one, the highest, exposure dose (250 mg/kg/d). Therefore, no reference value could be derived for PCB-180.

#### Overall study confidence ratings

A detailed summary of all risk of bias assessments and confidence ratings is shown in Table 1. Overall, eight of the 18 studies on individual PCB congeners were assigned to *TIER 1* (*High* confidence). These included one study investigating PCB-77, one study on PCB-118, three on PCB-126, two on PCB-153 and one testing PCB-169. The remaining ten studies were rated as *Medium* confidence (*TIER 2*), mainly because they had been rated as “definitely high risk” due to deficient reporting on funding sources or conflict of interest and an assessment of “probably high risk” due to the use of soy-based diet an in one case lack of information on the methods and timepoint for endpoint measurements. None of the studies were considered to be of *Low* confidence (*TIER 3*) since they all were rated at a sufficiently low risk in all key and other elements of the assessment.

#### Evidence synthesis

A summary of the study evaluations for all individual PCB congeners is shown in Table 2. Of the 18 observations, the majority described some adverse effect on selected semen quality parameters, while four studies reported no effects [39, 41, 45] and one study even observed an increase in daily sperm production [40].

We rated the overall evidence of an effect of PCB-77 on semen quality as *Slight*: One *TIER 1* [39] and one *TIER 2* study [42] showed declines in semen quality, but these effects were not seen in other studies [40, 41].

The evidence for declines in semen quality after PCB-118 exposure was assessed as *Robust.* The two available studies, one a *TIER 1* study [44], the other a *TIER 2* study [43], both reported disrupted sperm parameters.

The overall evidence for links between PCB-126 and deteriorations of semen quality is *Moderate*: Of the four available studies, two high confidence (*TIER 1*) studies observed a decrease in sperm counts. Due to low administered doses the effects in one study did not reach statistical significance [46], but significant effects were seen in a follow-up study with higher doses [47]. One *TIER 1* study [39] and one *TIER 2* study [45] did not demonstrate effects, but tested only one dose which may well have precluded detection of changed semen parameters.

The two *TIER 2* studies examining PCB-132 [48, 49] reported declines on semen quality. We did not identify additional *TIER 1* studies, but the evidence for declines in semen quality associated with PCB-132 exposures was consistent and we therefore ranked it as *Moderate.*

We identified only one study which tested PCB-149 [48]. This *TIER 2* study in rats described decreases in sperm quality, and accordingly, we considered the overall strength of evidence to be *Moderate*.

The two studies that examined PCB-153 exposures were rated as *high* confidence (*TIER 1*). One of them [45] was carried out in goats (see also PCB-126) and did not find any effects on semen quality. In this study only one dose of PCB-153 was tested, which was described as low dose. Thus, the absence of effects in this study is not conclusive. The second study was conducted in rats and found a decrease in daily sperm production [50]. Due to the clear effects in the *high* confidence study in rats, the explanation for the lack of effects in the goat study and in absence of further supporting or conflicting evidence, we considered the evidence for PCB-153 to be *Moderate*.

The effects of PCB-169 were investigated in two studies, one of overall *high* [51] and the second of *medium* [52] confidence. Both studies described declines in sperm counts. In the absence of conflicting evidence, the overall evidence for PCB-169 was regarded as *Robust*.

One study examined PCB-180 and found decreases in sperm counts in a subgroup of animals in the treatment group [53]. Although the study was of overall *medium* confidence, sperm counts were only assessed at the highest dose tested and the findings were equivocal. Therefore, in absence of additional studies, we consider the evidence for PCB-180 to be *Indeterminate*.

### Strength of evidence: human epidemiological studies

#### Study selection and evaluation

We identified 23 human epidemiological studies from the full text screening which were selected for data extraction and RoB assessment (Table 3). Most of these studies measured multiple PCB congeners, often in combination with other organochlorines or additional POPs. A few focused on single congeners, such as PCB-153 [71–76]. Combinations of PCBs-118, -138, -153 and -180 with other POPs were measured in six studies [77–82]. The remaining ten publications looked at a larger set of PCB congeners [83–93].

The ideal assessment of exposure to PCBs would be in maternal serum during pregnancy, as foetal development is a critical time period for semen quality in adulthood [94]. Only one of the eligible studies met these criteria, which measured PCB congeners in maternal serum, collected in pregnancy week 30 and semen quality in the sons (19–21 year old) [93].

In adult men, the duration of spermatogenesis is around 75 days plus an additional 12 days of maturation. Because PCBs bioaccumulate in fatty tissues, it is likely that existing exposures last over the entire period of spermatogenesis. The exposure assessment element in studies with a general description of sampling, extraction and analytical techniques was rated as “*adequate*” [72, 77, 82, 83, 87, 90–92]. The studies which provided detailed descriptions of quality assurance and analytical performance were evaluated as *“good”* with respect to the exposure aspect [71, 73–76, 78–81, 84–86, 88, 89, 93].

We assessed outcome measurement elements in relation to adherence to established quality standards described in the WHO guidelines [21]. These guidelines recommend the analysis of core semen parameters (number, concentration, motility and morphology). If all these parameters were analysed according to WHO standards, we evaluated the outcome measurement as *“good”* [72–76, 79–82, 84, 87, 88, 91, 93]. Studies which lacked details about the methods [92] or only reported sperm numbers [90] were rated as “*poor*”. All other studies, which conducted the outcome measurement according to WHO guidance, but did not provide all details on sampling and analysis or did not include sperm morphology measurements were evaluated as “*adequate*” [71, 77, 78, 83, 85, 86, 89].

Studies which selected participants from the general population with no apparent selection bias were rated as “*good*” [72–76, 84, 85, 93]. One study included infertile patients without control groups and was therefore evaluated as *"critically deficient”* [83]. One study provided limited information on participant selection and was rated as poor in relation to participant selection [89]. Another study which was part of a series of publications only referred to the description of the recruitment process in another publication was rated as “adequate/poor” [90]. The remaining studies were from fertility clinic or occupational settings and were classed as “*adequate*” [74, 77–82, 86, 88, 92].

We evaluated the quality of control for confounding by checking whether the following factors were accounted for: age, abstinence time, smoking history, body mass index and chronic disease status [95]. Alcohol use and stress could also be considered but are less well established. The majority of eligible studies took account of the key confounders and accordingly were rated as *“good”*. Where the key confounders were considered but some details were missing, we rated the study as *“adequate”* [72, 78, 81]. Studies which did not provide information on abstinence time were evaluated as “poor” [77, 79, 83, 89, 92].

When examining associations between declines in semen quality and exposure to PCBs, semen parameters should be analysed as continuous parameters to avoid misclassifications. Furthermore, sufficient detail should be provided, such as confidence intervals and standard errors, in addition to significance. Most of the studies fulfilled these criteria and were evaluated as “*good*” for data analysis. Weiss et al. did not provide sufficient detail on the analysis and did not show their data and was therefore rated as “*critically deficient*” [83]. If the data were dichotomised or some minor details on the analysis and results were not provided, the studies were rated as “adequate” [72, 73, 79–81, 87, 89, 90]. Studies with missing details to warrant an “*adequate*” rating were rated as *“poor*”[77, 82, 86, 92].

#### Overall study confidence ratings

We assigned overall study confidence ratings based on the ratings in the individual study evaluation elements, which are provided in Table 3. Of the 23 human epidemiological studies included in the analysis, ten studies had all or at least four of the evaluation aspects rated as *“good”* and one as *“adequate”*, and were assigned an overall *“High”* confidence rating. If two or three elements were rated as “good” and the remaining ones as *“adequate”* or maximally one as *“poor”*, as was the case in seven studies, we allocated an overall confidence of *“Medium”*. Four studies had two elements considered to be “poor” in addition to *“adequate”* or *“good”* ratings, and the overall confidence was pegged at a rating of *“Low”*. The remaining two studies had three or more *“poor”* ratings or were found to be *“critically deficient”*, and the overall confidence was classed as *“Uninformative”*.

#### Evidence synthesis

The outcomes of the 23 eligible epidemiological studies are summarised in Table 3. Nine studies reported null findings. One of these was judged to be *“Uninformative”* [83]. Two studies with null results were of “low” overall confidence [79, 90], two of “medium” confidence [82, 86] and four studies were of “high” confidence [84, 85, 91, 93].

Among the studies which reported effects, a diverse picture emerged. Four studies report mixed findings, with declines in semen quality for some PCB congeners or PCB metabolites, and improved semen parameters for other congeners in exposed populations compared to controls. One study which reported no effects for the congeners, declines in quality for metabolites and improvements for the sum of PCBs was rated as “low” confidence [77]. The study by Mumford et al. was of “medium” confidence and reported mix of declines or improvement for semen parameters, dependent on congener (Table 3) [87]. Two studies that found mixed results depending on congener and outcome measure were of “high” confidence [76, 88].

We identified three studies which only report improved semen parameters in exposed populations compared to controls for some parameters. Two of those were of “medium” confidence [78, 86] and one study was of “high” confidence [73].

The remaining eight studies all reported declines in semen quality for one or more parameters. One of these studies was considered to be “Uninformative” [92] and a second was judged to be “low” confidence [89]. We identified three “medium” confidence studies that reported declines in semen quality [72, 80, 81] and an additional three “high” confidence studies [71, 74, 75].

### Overall weight of evidence from human and experimental studies

There is *Robust* evidence from animal studies that PCB congeners -118 and -169 exposures lead to declines in semen quality. For congeners -126, -132 and -153 the evidence is *Moderate*. The evidence for PCB-77 from animal studies is only *Slight* and for PCB-180 the evidence was *Indeterminate*. In humans, only one study was available which measured PCB exposure during foetal life and assessed the semen quality in adults, and this study did not find any changes. Overall, the evidence from human epidemiological studies in adults is mixed and not all individual congeners have been examined. We did not identify human evidence for PCBs-77, -132, and 149. PCB-153 was investigated in several studies and the majority found declines in semen quality parameters, in line with the animal evidence, although studies reporting improved parameters do exist. One epidemiological study that included PCB-126 and another including PCB-169 supported the evidence from animal studies. For PCB-118 the human evidence was weak but generally in support of the animal studies. The evidence for PCB-180 from epidemiological studies was equivocal. Overall, the evidence from human studies is sufficiently robust to support hazard identification for some congeners and the commercial mixtures. We therefore used the evidence from animal studies to derive a reference dose for declines in semen quality for selected PCB congeners with sufficient evidence.

### Derivation of reference doses for declines in semen quality for PCB-118, -126, -132, -149, -153 and -169

We derived reference doses for PCB congeners with a *Moderate* or *Robust* evidence rating from animal studies and where there was no conflicting human evidence. Consequently, we estimated reference doses for PCB-118, -126, -132, -149, -153 and -169 (Table 4). PCB-77 and 180 were excluded as their confidence rating did not reach *Moderate*. Where studies reported data from three or more different dose groups (Table 4), we attempted BMD modelling to estimate a BMDL_5_. However, none of the selected studies provided adequate data and therefore we decided to use the NOAEL values as PoDs for all PCB congeners. Table 4 shows the PoDs derived from the studies which were included in the calculation of reference dose values.

#### PCB-118

One *TIER 1* study qualified for derivation of a reference dose for PCB-118 [44]. In this study PCB-118 was orally administered to mice during gestation (daily from GD 7.5 to GD 12.5). Two dose groups were exposed, and the authors reported a LOAEL of 20 µg/kg/d for declines in sperm with normal morphology. Using an AF of 3, we extrapolated a NOAEL of 6.67 µg/kg/d. By using the toxicokinetic parameters for PCB-118 ( t_1/2,a_ = 117 days, F_abs,a_ = 0.9 for the mouse and t_1/2,h_ = 3395 days, F_abs,h_ = 1 for the human) we first calculated the cumulative critical body burden at the NOAEL in the mouse before estimating the EHDI. The critical body burden was 35.5 µg/kg/d and the estimated EHDI was 0.00725 µg/kg/d. By applying the AF of 2.5, we derived reference dose value of 0.0029 µg/kg/d (Table 4).

#### PCB-126

The reference value for PCB-126 was derived from one *TIER 1* rat study which used 3 dose groups, and repeat administration from GD13 to GD19 [47]. The study determined a NOAEL of 0.25 µg/kg/d for declines in sperm numbers. With the kinetic parameters for PCB-126 ( t_1/2,a_ = 100 days, F_abs,a_ = 0.9 for the rat and t_1/2,h_ = 584 days, F_abs,h_ = 1 for the human) we estimated the critical body burden as 0.154 µg/kg/d and the corresponding EHDI as 0.00018 µg/kg/d. Applying the AF of 2.5 resulted in a reference dose value of 0.000073 µg/kg/d for PCB-126 (Table 4).

#### PCB-132

We identified two *TIER 2* rat studies which were eligible for inclusion in the derivation of a reference dose for PCB-132 [48, 49]. Both studies used a single i.p. administration in two dose groups, one during foetal development (GD15) [49] and in juvenile animals at PND 15 [48]. One of these studies reported a LOAEL of 1000 µg/kg/d for reductions in sperm numbers, which was extrapolated to a NOAEL of 333.33 µg/kg/d by using an AF of 3 [49]. The other study observed a higher LOAEL of 9600 µg/kg/d for declines in motility, which we extrapolated to a NOAEL of 3200 µg/kg/d [48]. Both studies used a single administration, thus, using an absorption of 90% in rodents, we calculated the critical body burden of PCB-132 at PoD by multiplying the NOAEL with the absorbed fraction, resulting in a body burden of 300 µg/kg/d [49] or 2880 µg/kg/d [48]. Applying the toxicokinetic parameters for PCB-132 (t_1/2,a_ = 100 days, F_abs,a_ = 0.9 for the rat and t_1/2,h_ = 3650 days, F_abs,h_ = 1 for humans) we calculated EHDI values of 0.057 µg/kg/d [49] and 0.547 µg/kg/d [48]. The reference doses were derived using an AF of 2.5, resulting in values of 0.0228 µg/kg/d [49] and 0.219 µg/kg/d [48]. The lower value derived from the gestational exposure study (0.0228 µg/kg/d) was chosen as reference dose for PCB-132 (Table 4).

#### PCB-149

The reference dose for PCB-149 was derived from the *TIER 2* study in juvenile rats which also tested PCB-132 [48]. The authors used a single i.p. administration at PND 15 and reported a NOAEL of 9600 µg/kg/d for reductions in sperm motility and velocity. Assuming 90% absorption, we calculated a critical body burden of 8640 µg/kg/d. With the toxicokinetic parameters for PCB-149 (t_1/2,a_ = 100 days, F_abs,a_ = 0.9 for the rat and t_1/2,h_ = 3650 days, F_abs,h_ = 1 for humans), we estimated an EHDI of 1.641 µg/kg/d. Using the AF of 2.5 we calculated the reference dose value of 0.656 µg/kg/d (Table 4).

#### PCB-153

We used one *TIER 1* rat study with two dose groups and repeat administration in pups (PND3) to derive a reference dose value for PCB-153 [50]. The study determined a NOAEL of 25 µg/kg/d for reductions in daily sperm productions as PoD. The PCB-153 toxicokinetic parameters ( t_1/2,a_ = 113 days, F_abs,a_ = 0.9 for the rat and t_1/2,h_ = 5256 days, F_abs,h_ = 1 for the human) were used to calculate the critical body burden in the animal (111 µg/kg/d) and the corresponding EHDI (0.0147 µg/kg/d). We applied the AF of 2.5 to derive the reference dose value of 0.00586 µg/kg/d (Table 4).

#### PCB-169

One *TIER 1* study in the rat was available to derive a reference dose for PCB-169 [51]. Using repeat oral dosing from PND1 to 7 in 3 dose groups, the authors reported a LOAEL of 25 µg/kg/d for decreases in sperm numbers and daily sperm production. We extrapolated the NOAEL (8.33 µg/kg/d) by applying an AF of 3. We estimated the critical body burden in the rat and the EHDI using the kinetic parameters for PCB-169 ( t_1/2,a_ = 85 days, F_abs,a_ = 0.9 for the mouse and t_1/2,h_ = 2665 days, F_abs,h_ = 1 for the human). The cumulative critical body burden had a value of 51.2 µg/kg/d, resulting in an EHDI of 0.0133 µg/kg/d. Finally, we applied the AF of 2.5 to account for differences between humans, resulting in a reference dose value of 0.00533 µg/kg/d for PCB-169 (Table 4).

### Comparison of reference doses with PCB exposures

To evaluate whether current exposures to specific PCB congeners exceed any of the above reference doses for deteriorations in semen quality, we used exposure data from the European Union.

The average exposures of European adults to PCB-169 via food are around 0.00079 ng/kg/d, but these can increase to 0.0024 ng/kg/d (mean and 95^th^ percentile LB intake for adults, calculated from the percentage contribution of individual congeners to sums of dl-PCBs [2]). Both these values are far below the reference dose of 5.33 ng/kg/d (Table 5). For PCB-126, the average exposures via food are around 0.0035 ng/kg/d, with high levels rising to 0.01 ng/kg/d [2]. Whereas the average value is well below the reference dose of 0.073 ng/kg/d, the high exposure is less than an order of magnitude below the reference dose, resulting in a Risk Quotient of 0.14 (Table 5). Average exposures to PCB-118 via food are around 0.576 ng/kg/d, with high exposures up to 1.7 ng/kg/d [2]. Both these values are relatively close to the reference dose of 2.9 ng/kg/d, resulting in Risk Quotients of 0.2 and 0.59 respectively (Table 5). We did not identify exposure levels for PCB-132, -149, or -153. PCB-153 is frequently assessed as part of the sum of 6 indicator PCBs, which also includes PCB-118. PCB levels in food are highly correlated and PCB-153 is often present at levels up to three times higher than PCB-118 [3]. Thus, as a worst-case assumption average and high exposures to PCB-153 via the diet could be estimated to be around 1.7 ng/kg/d and 5.1 ng/kg/d respectively (Table 5). This would also put the exposures close to the reference value of 5.86 ng/kg/d with Risk Quotients of 0.29 and 0.87 for average and high exposures, respectively. No exposures for PCB-132 and -149 could be retrieved, however, these congeners are not part of common indicator PCB groups and are with their higher reference doses of 22.8 ng/kg/d (PCB-132) and 656 ng/kg/d (PCB-149) likely of lower concern.

**Table 5:** Calculation of Risk Quotients for individual PCB congeners

PCB congener	RfD	Average consumption		High consumption	
		**Exposure** **(ng/kg/d)**	**Risk Quotient** **average**	**Exposure** **(ng/kg/d)**	**Risk Quotient** **high**
PCB-118	2.9	0.576	0.2	1.7	0.59
PCB-126	0.073	0.0035	0.05	0.01	0.14
PCB-153	5.86	1.7	0.29	5.1	0.87
PCB-169	5.33	0.00079	0.00015	0.0024	0.00045

*RfD* Reference dose

The overall HI for PCB-118, -126, -153 and -169 for average exposures observed in European adults would be 0.54, relatively close to the value of 1. For the higher exposure scenario, the HI is 1.58 and therefore exceeding the index value of 1.

## Discussion

Mixture risk assessments require reference doses derived from toxicity data for a common health endpoint. To assess mixture risks for male reproductive health, we chose declines in semen quality associated with chemical exposures as the specific endpoint. Although PCBs are usually used in technical mixtures which contain several congeners, it was necessary to derive reference doses for individual PCB-congeners to derive the Risk Quotients required for the mixture risk assessment. It would not be feasible to derive the Risk Quotient for technical PCB mixtures due to the unknown specific composition used and the uncertainty which of the PCB congers within the mixtures reach human tissues. Here we derived reference doses for the PCB congeners PCB-118, -126, -132, -149, -153 and -169, for which we considered the evidence for deteriorations of semen quality as sufficiently strong. For PCB-77 and -180 the evidence was not strong enough to derive a reference dose. However, considering the majority of animal studies with PCB mixtures and the evidence from human epidemiological studies included in this review, there is clear evidence that exposure to the PCB congeners which were used to derive reference doses and to PCB mixtures can interfere with semen quality parameters. Whilst it is not known what might cause the “improvements” in semen quality seen in some studies, these observations were usually made while other parameters evaluated at the same time indicated adverse effects, such as interference with hormone levels or impaired development of male reproductive organs.

In support of the findings that PCBs can adversely affect semen quality, the technical PCB mixture Aroclor 1254 is commonly used in animal studies specifically to induce declines in semen quality with the aim of examining the effects of therapeutic or preventative treatments.

The optimal exposure timing for detecting declines in semen quality is the critical developmental period when germline stem cell populations are established (GD7 to PND 8 in the mouse and GD 9 to PND 10 in rats). However, when gestational studies were not available, we also considered data from juvenile or adult animals. As a basis for deriving reference doses, we used studies where PCBs were administered during gestation, perinatal life or to juvenile animals. The only congener for which data from prenatal and juvenile exposures had to be used was PCB-132 [48, 49]. The prenatal exposures resulted in an approximately ten-fold lower reference dose, suggesting that exposures during gestation have a greater impact on semen quality.

When deriving reference doses, we did not consider data from animal studies that used Aroclor mixtures, due to the uncertainties regarding their composition. Epidemiological studies were used as supplementary evidence for associations with deteriorations of semen quality in humans but were not included to derive a reference dose.

Although we adhered to commonly used risk assessment practices in deriving reference doses for declines in semen quality [2, 96], our values do not have the normative character of HBGV and are only intended for the purpose of mixture risk assessment for male reproductive toxicity. They should be taken as “reasonable” potency estimates for this kind of toxicity. Some of the animal studies we had to use for our estimates fall short of the standards required for deriving HBGV in terms of study quality and data demands such as number of doses, animals and reporting. Furthermore, declines in semen quality may not always represent the critical toxicity of PCB congeners and therefore the HBGV for the individual compound would be lower than the reference values derived here. For all these reasons, the values proposed here should not be used in the context of chemical risk assessments for individual congeners. Mixing references doses for different toxicities in a mixture risk assessment would overestimate the mixture risks, and increase the uncertainty of the assessment. Using endpoint specific reference doses increases the confidence in the mixture risk assessment, even if the underlying data is not of the highest quality. In cases were chemicals based on lower quality data would become drivers of mixture risk due to a high Risk Quotient, these chemicals than should be prioritised for further investigation.

Due to their persistence, PCBs are still found in the environment and human tissues, despite not being in use for some time since their ban several decades ago. Thus, humans are still exposed to PCBs, mainly via food and estimates for dietary exposure to several PCB congeners have been reported [2]. We compared the reference doses with human dietary exposures where these were available, i.e. PCB-118, -126, -153 and -169. None of the Risk Quotients for individual PCB-congeners exceeded the value of 1, neither for average, nor for high exposure scenarios. However, the sum of Risk Quotients, i.e. the HI, for all four congeners at average exposures was 0.54. For high exposures, this sum was 1.6, in exceedance of the value 1. Therefore, PCBs as a group may already on their own pose a mixture risk in certain exposure scenarios. Using the HI to estimate the mixture risk assumes that the effect is dose additive and no interactions such as synergism or antagonism occur. Whilst synergisms are of particular concern, they are rare and commonly involve specific classes of compounds [97]. We therefore consider dose addition as a suitable default assumption for a mixture risk assessment of male reproductive health. Several other chemicals, such as phthalates, bisphenols, some PBDE congeners, certain pesticides and analgesics, are known to cause deterioration in semen quality [8]. We have previously established reference doses for BPA and PBDEs for declines in semen quality [29, 30] to be used together with the values for PCB congeners established in this study in a mixture risk assessment for this endpoint.

## Supplementary Information

**Additional file 1: Supplementary Table 1.** PECO statement for animal studies. **Supplementary Table 2.** PECO statement for human studies. **Supplementary Table 3.** Eligibility criteria for animal studies. **Supplementary Table 4.** Eligibility criteria for human studies. **Supplementary Table 5.** Key data extraction elements to summarise study design, experimental model, methodology and results. **Supplementary Table 6.** Toxicokinetic parameters for PCB-118, -126, -132, -149, -153 and -169.

## Data Availability

Not Applicable.

## Abbreviations

AF: Assessment factor
AhR: Aryl hydrocarbon receptor
AR: Androgen receptor
BMDL: Benchmark dose level
BPA: Bisphenol A
COSTER: Conduct of Systematic Reviews in Toxicology and Environmental Health Research
dl-PCB: Dioxin like-PCB
ECHA: European Chemicals Agency
EDC: Endocrine disrupting chemical
EFSA: European Food Safety Authority
EHDI: Estimated human daily intake
GD: Gestational day
HBGV: Health-based guidance value
HI: Hazard Index
i.p.: Intraperitoneal
LOAEL: Lowest observed adverse effect level
ndl-PCB: Non-dioxin-like PCB
NOAEL: No observed adverse effect level
PBDE: Polybrominated diphenyl ether
PCB: Polychlorinated biphenyl
PCDD: Polychlorinated dibenzodioxin
PCDF: Polychlorinated dibenzofuran
PND: Postnatal day
PoD: Point of departure
POP: Persistent organic pollutant
RoB: Risk of Bias
RPF: Relative potency factor
s.c.: Subcutaneous
TEQ: Toxic equivalent
TWI: Tolerable weekly intake
