# Supplementary material for: Systematic review of associations of polychlorinated biphenyl (PCB) exposure with declining semen quality in support of the derivation of reference doses for mixture risk assessments
Source: Environ Health. 2022 Oct 11;21:94. doi: 10.1186/s12940-022-00904-5 (PMC9552438; doi:10.1186/s12940-022-00904-5)
Supplement: Supplementary file 1 — Additional file 1: Supplementary Table 1. PECO statement for animal studies. Supplementary Table 2. PECO statement for human studies. Supplementary Table 3. Eligibility criteria for animal studies. Supplementary Table 4. Eligibility criteria for human studies. Supplementary Table 5. Key data extraction elements to summarise study design, experimental model, methodology and results. Supplementary Table 6. Toxicokinetic parameters for PCB-118, -126, -132, -149, -153 and -169. [file 12940_2022_904_MOESM1_ESM.pdf]

Systematic review of associations of polychlorinated biphenyl (PCB) exposure with declining semen quality in support of the derivation of reference doses for mixture risk assessments

Sibylle Ermler* and Andreas Kortenkamp

Brunel University London
Centre for Pollution Research and Policy
College of Health, Medicine and Life Sciences
Kingston Lane
Uxbridge UB8 3PH
United Kingdom

Email: sibylle.ermler@brunel.ac.uk
* address correspondence: Sibylle Ermler

ORCID:
Sibylle Ermler: https://orcid.org/0000-0002-2626-9548
Andreas Kortenkamp: https://orcid.org/0000-0001-9055-9729

Supplementary Material S1

Supplementary Table 1. PECO statement for animal studies

Supplementary Table 2. PECO statement for human studies

Supplementary Table 3. Eligibility criteria for animal studies

Supplementary Table 4. Eligibility criteria for human studies

Supplementary Table 5. Key data extraction elements to summarise study design, experimental model, methodology and results

Supplementary Table 6. Toxicokinetic parameters for PCB-118, -126, -132, -149, -153 and -169

Supplementary Table 7. PECO statement for animal studies

Question	Is exposure to PCBs associated with declines in semen quality?
**Populations**	Laboratory mammalian species including rats, mice, rabbits, guinea pigs, dogs, sheep and monkeys
**Exposures**	PCBs by oral gavage, via drinking water or the diet during gestation and postnatal life, when germ cell populations are established (gestational day 7 to postnatal day 8 in mice; gestational day 9 to postnatal day 10 in rats).
**Comparators**	Animals not exposed to PCBs
**Outcomes**	Semen quality, as measured in terms of:<br>• Total sperm count<br>• Sperm concentration<br>• Sperm motility<br>• Sperm morphology<br>• Sperm vitality

Supplementary Table 8. PECO statement for human studies

Question	Is exposure to PCBs associated with declines in semen quality?
**Populations**	Men of reproductive age (between 18 and 40 years of age)
**Exposures**	PCBs, measured as blood, serum or plasma levels in expectant mothers or at time points close to the collection of semen samples in adult men
**Comparators**	Men not exposed to PCBs, or men with PCB levels in lower quartiles
**Outcomes**	Semen quality, as measured in terms of:<br>• Total sperm count<br>• Sperm concentration<br>• Sperm motility<br>• Sperm morphology<br>• Sperm vitality

Supplementary Table 9. Eligibility criteria for animal studies

		Inclusion criteria	Exclusion criteria
**Populations**	Laboratory mammalian species including rats, mice, rabbits, guinea pigs, dogs and monkeys	Mammalian species	Non mammalian test species such as fish or amphibians
**Exposures**	PCB perinatally, e.g. at any time from gestational day 7 (mouse) or 9 (rat) to postnatal day 8 (mouse) or 10 (rat) preferred.<br>Absent gestational studies, data from juvenile or adult animals were considered.	Administered by gavage, via drinking water or through the diet; at least 2 exposure doses.<br>Intraperitoneal administration was considered if no oral data was available.	Administered subcutaneously*; only 1 exposure dose group; in juveniles or adults.<br><br>(*s.c. was considered as supporting evidence but not for deriving a reference dose)
**Comparators**	Animals not exposed to PCBs	Control group (same species as exposure group(s))	No control group
**Outcomes**	Semen quality	• Total sperm count<br>• Sperm concentration<br>• Sperm motility<br>• Sperm morphology<br>• Sperm vitality	• Sperm DNA damage<br>• Aneuploidies<br>• Fertility and fertilization outcomes

Supplementary Table 10. Eligibility criteria for human studies

		Inclusion criteria	Exclusion criteria
**Populations**	Men	Younger than 40 years, older than 18 years	Men with non-descended testes, hypospadias, and chronic diseases such as cancer, varicocele, or other known illnesses impacting on semen quality; men > 40 years; < 18 years
**Exposures**	PCB	PCB exposures measured as blood, serum or plasma concentrations.	PCBs in other body fluids or tissues, e.g. adipose tissue or seminal fluid, exposure information derived from questionnaires or job exposure matrices
**Comparators**	Exposure contrast PCB lower levels or in reference groups	Sufficient information reported to allow comparison/categorisation of exposures.	Insufficient information reported to allow comparison/categorisation of exposures.
**Outcomes**	Semen quality	• Total sperm count<br>• Sperm concentration<br>• Sperm motility<br>• Sperm morphology<br>• Sperm vitality	• Sperm DNA damage<br>• Aneuploidies<br>• Measures of in vitro fertilization success<br>• Time to pregnancy
**Design**		• Case-control studies<br>• Cohort studies<br>• Cross-sectional studies	• Case reports<br>• Reviews

Supplementary Table 11. Key data extraction elements to summarise study design, experimental model, methodology and results (Reproduced from [1])

**HUMAN**	
Funding	Funding source(s)
	Reporting of conflict of interest (COI) by authors (*reporting bias)
Subjects	Study population name/description
	Dates of study and sampling time frame
	Geography (country, region, state, etc.)
	Demographics (sex, race/ethnicity, age or lifestage at exposure and at outcome
	Number of subjects (target, enrolled, n per group in analysis, and
	Inclusion/exclusion criteria/recruitment strategy (*selection bias)
	Description of reference group (*selection bias)
Methods	Study design (e.g., prospective or retrospective cohort, nested case-control study, cross-sectional, population-based case-control study, intervention, case report,
	Length of follow-up (*information bias)
	Health outcome category, e.g., cardiovascular
	Health outcome, e.g., blood pressure (*reporting bias)
	Diagnostic or methods used to measure health outcome (*information bias)
	Confounders or modifying factors and how considered in analysis (e.g., included in final model, considered for inclusion but determined not needed
	Substance name and CAS number
	Exposure assessment (e.g., blood, urine, hair, air, drinking water, job classification, residence, administered treatment in controlled study, etc.)
	Methodological details for exposure assessment (e.g., HPLC-MS/MS, limit of
	Statistical methods (*information bias)
Results	Exposure levels (e.g., mean, median, measures of variance as presented in paper, such as SD, SEM, 75th/90th/95th percentile, minimum/maximum); range of [illegible]
	Statistical findings (e.g., adjusted β, standardized mean difference, adjusted odds ratio, standardized mortality ratio, relative risk, etc.) or description of qualitative results. When possible, OHAT will convert measures of effect to a common metric with associated 95% confidence intervals (CI). Most often, measures of effect for continuous data are expressed as mean difference, standardized mean difference, and percent control response. Categorical data are typically expressed as odds ratio, relative risk (RR, also called risk ratio), or β values, depending on
	If not presented in the study, statistical power can be assessed during data extraction using an approach that can detect a 10% to 20% change from response by control or referent group for continuous data, or a relative risk or odds ratio of 1.5 to 2 for categorical data, using the prevalence of exposure or prevalence of outcome in the control or referent group to determine sample size. For categorical data where the sample sizes of exposed and control or referent groups differ, the sample size of the exposed group will be used to determine the relative power category. Recommended sample sizes to achieve 80% power for a
	Observations on dose response (e.g., trend analysis, description of whether dose-response shape appears to be monotonic, non-monotonic)
Other	Documentation of author queries, use of digital rulers to estimate data values from figures, exposure unit, and statistical result conversions, etc.

ANIMAL	
Funding	Funding source(s)
	Reporting of COI by authors (*reporting bias)
Animal Model	Sex
	Species
	Strain
	Source of animals
	Age or lifestage at start of dosing and at health outcome assessment
	Diet and husbandry information (e.g., diet name/source)
Treatment	Chemical name and CAS number
	Source of chemical
	Purity of chemical (*information bias)
	Dose levels or concentration (as presented and converted to mg/kg bw/d when
	Other dose-related details, such as whether administered dose level was verified by measurement, information on internal dosimetry (*information bias)
	Vehicle used for exposed animals
	Route of administration (e.g., oral, inhalation, dermal, injection)
	Duration and frequency of dosing (e.g., hours, days, weeks when administration
Methods	Study design (e.g., single treatment, acute, subchronic (e.g., 90 days in a rodent), chronic, multigenerational, developmental, other)
	Guideline compliance (i.e., use of EPA, OECD, NTP or another guideline for study design, conducted under GLP guideline conditions, non-GLP but consistent with
	Number of animals per group (and dams per group in developmental studies)
	Randomization procedure, allocation concealment, blinding during outcome
	Method to control for litter effects in developmental studies (*information bias)
	Use of negative controls and whether controls were untreated, vehicle-treated, or
	Report on data from positive controls – was expected response observed?
	Endpoint health category (e.g., reproductive)
	Endpoint (e.g., infertility)
	Diagnostic or method to measure endpoint (*information bias)
	Statistical methods (*information bias)
Results	Measures of effect at each dose or concentration level (e.g., mean, median, frequency, and measures of precision or variance) or description of qualitative results. When possible, OHAT will convert measures of effect to a common metric with associated 95% confidence intervals (CI). Most often, measures of effect for continuous data will be expressed as mean difference, standardized mean
	No Observed Effect Level (NOEL), Lowest Observed Effect Level (LOEL), benchmark dose (BMD) analysis, statistical significance of other dose levels, or other estimates of effect presented in paper. Note: The NOEL and LOEL are highly influenced by study design, do not give any quantitative information about the relationship between dose and response, and can be subject to author's interpretation (e.g., a statistically significant effect may not be considered
	If not presented in the study, statistical power can be assessed during data extraction using an approach that assesses the ability to detect a 10% to 20% change from control group's response for continuous data, or a relative risk or odds ratio of 1.5 to 2 for categorical data, using the outcome frequency in the control group to determine sample size. Recommended sample sizes to achieve 80% power for a given effect size, i.e., 10% or 20% change from control, will be
	Observations on dose response (e.g., trend analysis, description of whether dose-response shape appears to be monotonic, non-monotonic)
	Data on internal concentration, toxicokinetics, or toxicodynamics (when reported)
Other	Documentation of author queries, use of digital rulers to estimate data values from figures, exposure unit, and statistical result conversions, etc.

Supplementary Table 12. Toxicokinetic parameters for PCB-118, -126, -132, -149, -153 and -169

	PCB-118	PCB-126	PCB-132	PCB-149	PCB-153	PCB-169
$t_{1/2,a}$	117	100	100	100	113	85
$t_{1/2,h}$	3395	584	3650	3650	5256	2665
$F_{abs,a}$	0.9	0.9	0.9	0.9	0.9	0.9
$F_{abs,h}$	1	1	1	1	1	1

All kinetic parameters were collected from EFSA [2,3] or published literature [4–7].

$t_{1/2,a}$ = halflife of excretion in animals (in days)

$t_{1/2,h}$ = halflife of excretion in humans (in days)

$F_{abs,a}$ = Fraction of chemical absorbed into the animal body

$F_{abs,h}$ = Fraction of chemical absorbed into the human body

References

1. NTP OHAT. Handbook for Conducting a Literature-Based Health Assessment Using OHAT Approach for Systematic Review and Evidence Integration; March 4, 2019. 2019;

2. EFSA. Risk for animal and human health related to the presence of dioxins and dioxin-like PCBs in feed and food. EFSA J. 2018;16.

3. EFSA. Scientific statement on the health-based guidance values for dioxins and dioxin-like PCBs. EFSA J. 2015;13.

4. O'Grady Milbrath M, Wenger Y, Chang CWCW, Emond C, Garabrant D, Gillespie BW, et al. Apparent half-lives of dioxins, furans, and polychlorinated biphenyls as a function of age, body fat, smoking status, and breast-feeding. Environ Health Perspect. 2009;117:417–25.

5. Ogura I. Half-life of each dioxin and PCB congener in the human body. Organohalogen Compd. 2004;66:3329–37.

6. Öberg M, Sjödin A, Casabona H, Nordgren I, Klasson-Wehler E, Håkansson H. Tissue distribution and half-lives of individual polychlorinated biphenyls and serum levels of 4-hydroxy-2,3,3′,4′,5-pentachlorobiphenyl in the rat. Toxicol Sci. 2002;70:171–82.

7. Ritter R, Scheringer M, MacLeod M, Moeckel C, Jones KC, Hungerbühler K. Intrinsic human elimination half-lives of polychlorinated biphenyls derived from the temporal evolution of cross-sectional biomonitoring data from the United Kingdom. Environ Health Perspect. 2011;119:225–31.

8. Xiao W, Zhang J, Liang J, Zhu H, Zhou Z, Wu Q. Adverse effects of neonatal exposure to 3,3′,4,4′,5,5′-hexachlorobiphenyl on hormone levels and testicular function in male Sprague-Dawley rats. Environ Toxicol. School of Public Health, Fudan University, P.O. Box 288, 130 DongAn Road, Shanghai 200032, China; 2011;26:657–68.
